# Targeted protein delivery: carbodiimide crosslinking influences protein release from microparticles incorporated within collagen scaffolds

**DOI:** 10.1093/rb/rbz015

**Published:** 2019-04-22

**Authors:** Constantin Edi Tanase, Omar Qutachi, Lisa J White, Kevin M Shakesheff, Andrew W McCaskie, Serena M Best, Ruth E Cameron

**Affiliations:** 1 Department of Materials Science and Metallurgy, University of Cambridge, Cambridge Centre for Medical Materials, Cambridge, 27, Charles Babbage Road, UK; 2 Centre for Biomolecular Sciences, School of Pharmacy, University of Nottingham, University Park, Nottingham, UK; 3 Division of Trauma & Orthopaedic Surgery, Department of Surgery, University of Cambridge, Addenbrooke’s Hospital, Hills Road, Cambridge, UK

**Keywords:** collagen scaffolds, PLGA microparticles, FITC-BSA, EDC crosslinking, pore size, percolation diameter

## Abstract

Tissue engineering response may be tailored via controlled, sustained release of active agents from protein-loaded degradable microparticles incorporated directly within three-dimensional (3D) ice-templated collagen scaffolds. However, the effects of covalent crosslinking during scaffold preparation on the availability and release of protein from the incorporated microparticles have not been explored. Here, we load 3D ice-templated collagen scaffolds with controlled additions of poly-(DL-lactide-co-glycolide) microparticles. We probe the effects of subsequent *N*-(3-dimethylaminopropyl)-*N*′-ethylcarbodiimide hydrochloride crosslinking on protein release, using microparticles with different internal protein distributions. Fluorescein isothiocyanate labelled bovine serum albumin is used as a model protein drug. The scaffolds display a homogeneous microparticle distribution, and a reduction in pore size and percolation diameter with increased microparticle addition, although these values did not fall below those reported as necessary for cell invasion. The protein distribution within the microparticles, near the surface or more deeply located within the microparticles, was important in determining the release profile and effect of crosslinking, as the surface was affected by the carbodiimide crosslinking reaction applied to the scaffold. Crosslinking of microparticles with a high proportion of protein at the surface caused both a reduction and delay in protein release. Protein located within the bulk of the microparticles, was protected from the crosslinking reaction and no delay in the overall release profile was seen.

## Introduction

Tissue engineering approaches have emerged as potential therapeutic strategies for the repair of the soft tissue loss due to trauma and disease [1–7], with most involving the use of three-dimensional (3D) scaffolds with controlled architecture and biological response [8–12]. A successful 3D scaffold requires optimal mechanical properties and porous architecture in addition to a controlled cell-substrate interaction, low inflammatory response and a controllable degradation rate. Collagen, as a major constituent of the extracellular matrix, is widely used as a base material [13–17] and may be ice-templated to create open porous structures [10, 18, 19]. By controlling the ice nucleation and growth, one can achieve tuneable architectural structures with defined pore sizes and transport pathways [10, 20, 21]. Scaffold architecture can be affected by the mould design, which can influence the nucleation and growth of ice [22]. The mechanical properties and stability of the 3D ice-templated collagen scaffolds are important for cell-biomaterial interaction and, while dependent on the scaffold composition, are routinely controlled by applying crosslinking processes to the scaffold [18, 19, 23].

The most common methods used for crosslinking collagen-based scaffolds are ultraviolet (UV) irradiation, dehydrothermal treatment and *N*-(3-Dimethylaminopropyl)-*N*′-ethylcarbodiimide hydrochloride (EDC) treatment. UV and dehydrothermal treatment can destroy the native structure of the protein inducing fragmentation of the molecular structure, denaturation of the protein and increasing degradation rate [24–26]. EDC is a ‘zero length’ crosslinking agent and along with its by-product (urea) can be easily removed by washing [27–30]. The use of the EDC method involves a coupling reaction of free amino groups and carboxylate anions.

Ice-templated collagen scaffolds have wide potential application in the biomedical field including in the treatment of articular lesions [7, 16, 17, 31, 32], and it would be of further benefit if they were able to deliver specific and targeted biomolecules, over a sustained period, to optimize and refine the therapeutic response. Poly-(DL-lactide-co-glycolide) microparticles (PLGA) systems can offer a controllable release of the therapeutic agents [33] with complete resorbability [34–37].

The incorporation of nanoparticles/microparticles/microspheres into 3D ice-templated scaffolds and the consequent protein release behaviour has been previously reported [38–44]. However, the effects of the microspheres on the final scaffold architecture and the effect of the crosslinking agent on the bovine serum albumin-fluorescein isothiocyanate conjugate (FITC-BSA) drug release was not evaluated, 3D pore size distribution and percolation diameter were not assessed and the effect of EDC crosslinking on release kinetics of the incorporated drug model was not considered, although there is evidence that EDC can affect release in other contexts[45–47].

It is clear that the effect of the addition microparticles on the structure and accessibility of ice-templated scaffolds are little understood. These effects are important because pore size and percolation diameter are key factors affecting the cell invasion and nutrient supply [48]. Furthermore, many applications require the stiffness and stability achieved via EDC crosslinking [11, 19, 23], without the disadvantages inherent in other crosslinking methods. However, the effects of EDC crosslinking on protein release from microparticles loaded in 3D ice-templated scaffolds have not been reported.

In this article, we explore the effects of FITC-BSA-loaded microparticles addition on the structure and transport pathways within a 3D ice-templated collagen scaffold using microCT and percolation analysis. We further investigate scaffolds in which the added microparticles are of similar size but have very different internal drug distributions, to explore the effect of EDC crosslinking reactions on protein release.

## Materials and methods

### Materials reagents and chemicals

Insoluble fibrillar Type I collagen from bovine Achilles tendon CAS # C9879, phosphate buffered saline (PBS) tablets CAS # P4417, *N*-(3-Dimethylaminopropyl)-*N*′-ethylcarbodiimide hydrochloride (EDC) CAS # E1769, N-hydroxysuccinimide (NHS) CAS # 130672, ethanol (96% V/V) and FITC-BSA CAS # A9771, were purchased from Sigma-Aldrich, UK. Poly-(lactic-co-glycolic acid) (PLGA 85:15 DLG 5A) with acid end group, item code PS5126a.5, was obtained from Lakeshore Biomaterials (formerly Lakeshore Biomaterials), Birmingham, AL, USA. Acetic acid was obtained from Alfa Aesar, UK.

### Microparticles fabrication and characterization

Two batches of PLGA 85:15 microparticles were assessed in this study, named MP-1 and MP-2. PLGA 85:15 microparticles were prepared as previously described [36, 49]. Microparticles were fabricated from 20% PLGA in dichloromethane (DCM, Fisher, UK) by the double emulsion method. The polymer solution plus aqueous solution of deionized water containing FITC-BSA (Sigma Aldrich, UK) were homogenized using a high-speed Silverson L5M homogenizer (Silverson Machines, UK). For MP-1, 1 g of PLGA dissolved in 5 ml DCM was homogenized with 10 mg FTIC-Albumin dissolved in 100 µl of deionized water. For MP-2, 2 g of PLGA dissolved in 10 ml DCM was homogenized with 20 mg FTIC-Albumin dissolved in 200 µl of deionized water. The resultant primary water in oil (w/o) emulsion was then homogenized again in 0.3% polyvinyl alcohol (Sigma Aldrich, UK) and the resultant water in oil in water (w/o/w) double emulsion was left stirring at 300 rpm until the microparticles hardened and then harvested. To obtain release profiles from the PLGA 85:15 microparticle batches, 25 mg microparticles from MP-1 and MP-2 were dispersed into 1.5 ml PBS in microtubes. The microtubes were placed in an incubator at 37°C under shaking at 10 rpm for the entire duration of the experiment (e.g. 59 days). About 100 μl from the supernatant was collected at the prescribed time points and analysed for FITC-BSA content. The same volume of fresh PBS was replenished into the microtubes, simulating the constant dissipation of the released drug inside the body.

Microparticles were characterized in terms of surface morphology, mean microparticle diameter and microparticle size distribution. Characterization of surface morphology was undertaken using scanning electron microscopy (SEM). The microparticles were loaded onto carbon discs (Agar Scientific, UK) mounted on aluminium stubs (Agar Scientific, UK). The microparticles were gold-coated using Balzers SCD030 gold sputter coater (Balzers Union Ltd., Liechtenstein). Imaging of the microparticles was done using JEOL 6060L scanning electron microscope imaging system (JEOL Ltd., Hertfordshire, UK). The mean diameter and microparticle size distribution were also investigated using Coulter LS230 microparticle size analyser (Beckman, UK). Microparticle size distribution was then determined as a function of the microparticle diffraction and analysed as a function of volume percentage.

Microparticles from each batch were analysed by confocal laser scanning microscopy (CLSM) carried out on 270 an Olympus FV1200 microscope (Olympus, JP) to study the distribution of the FITC-BSA within them.

### Fabrication of 3D ice-templated collagen scaffolds loaded with PLGA 85:15 microparticles

**Figure 1:**
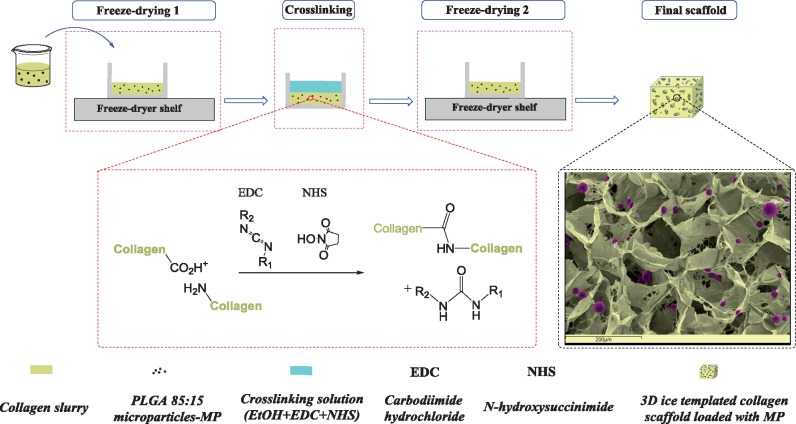
Schematic stages of producing 3D ice-templated collagen scaffolds loaded with PLGA 85:15 microparticles. The SEM image uses false colour.

The 3D ice-templated collagen scaffolds were obtained as previously described [19, 50]. Briefly, collagen suspension (1% w/v) was achieved by hydrating insoluble fibrillar Type I collagen in 0.05 M acetic acid for 72 h. After homogenization and removal of air bubbles, PLGA 85:15 microparticles were mixed with the collagen slurry at levels of 3, 5, 11, 22 and 44 mg⋅ml^−1^. The resulting slurries were poured into six-well plates (Thermo Scientific, UK). Freeze-drying was carried out with a VirTis advantage benchtop freeze-drier (BioPharma Process Systems, UK), using a constant cooling rate of 1°C⋅min^−1^ to a final freezing temperature of −20°C. The temperature was held for 200 min to ensure freezing was complete, at which point the ice was sublimed under a vacuum of 80 mTorr at a temperature of 0°C, maintained for 20 h. Figure 1 summarizes the stages of the scaffold manufacture process.

### Scaffold stabilization

The 3D ice-templated collagen scaffolds were crosslinked using a molar ratio of EDC to NHS to COOH of 0.5:0.2:1 using ethanol (95% v/v) as solvent. This was defined as a 10% crosslinking condition [51]. After incubation for 2 h at room temperature, the scaffolds were washed extensively with deionized water before freeze-drying once again using the same freeze-drying protocol as described above. The microstructure of the 3D ice-templated collagen scaffolds with and without the PLGA 85:15 microparticles was analysed by SEM (JEOL 5800, JEOL, Welwyn Garden City, UK). Gold-coated samples were imaged at 10 kV.

### X-ray micro-computed tomography

Quantitative X-ray micro-computed tomography (μCT) analysis was performed using a Skyscan 1172 (Bruker, Belgium) with a pixel size of 2.44 μm. The scans were taken at 25 Kv and 137 μA, using a 0.2° step size and a frame average of 2 and 180° rotation. Skyscan’s NRecon software was used to reconstruct the raw shadow projections into 3D data sets (version 1.6.9.8 Bruker, Belgium). Three cubic regions of interest (ROIs) were defined within each scaffold (1 mm^3^) to allow investigation of structure. All the analysis was carried out using CTAn software (version 1.17.7.2 Bruker, Belgium). 3D pore analysis was carried out in CTAn [52]. The CTAn software was also used for calculation of percolation diameter as described previously by Ashworth *et al*. [53] using CTAn’s shrink wrap feature by identifying the accessible volume of a virtual object that infiltrates through entire ROI. Briefly, by increasing the diameter ‘*d*’ the virtual object the corresponding length of the accessible pore volume ‘*l*’ in *z* direction can be measured using the relationship:

$$L=L_{0}{\left(d-d_{c}\right)}^{-\&thetasym;}$$

where ϑ  is a percolation constant equal to 0.88 for 3D systems [54].

The total number of PLGA 85:15 microparticles within a 3D ice-templated collagen scaffold was calculated from a 2.44 μm pixel size 3D data set using CTAn software. Briefly, 1 mm^3^ ROI were used to assess the number of microparticles per unit volume. The images were threshold and binarized followed by an individual 3D object analysis using CTAn software.

### *In vitro* FITC-BSA release profiles from 3D ice-templated collagen scaffolds

Release profiles were measured in similar condition as described for microparticles batches (MP-1 and MP-2). Briefly, the 3D ice-templated collagen scaffolds loaded with PLGA 85:15 microparticles (MP-1 and MP-2) were placed in PBS at 37°C under shaking at 10 rpm. The 5 mm diameter samples from 3D ice-templated collagen scaffolds for each condition MP-1 and MP-2 loaded at 22 mg⋅ml^−1^ were placed in microtubes. At each time point (1, 3, 4, 7, 10, 15, 21, 31, 43 and 59 days) 100 μl of the buffer was removed and stored at −20° C until further analysis. A fresh 100 μl of the buffer was added to the samples and they were re-incubated until the following time point. The amount of the FITC-BSA released was quantified measured by fluorescence read using a FLUOstar Optima (BMG Labtech, Offenburg, Germany) plate reader at 485 nm excitation, 520 nm emission. The concentration of the FITC-BSA was calculated by comparison to the standard curve of FITC-BSA. Six replicates for each condition were used, and the data points are shown as mean ± standard deviation (SD).

### Statistical analysis

Statistical significance was calculated using one-way analysis of variance. All statistical analysis was carried out using the standard statistics package in Origin Pro 2015 (Origin Lab, USA).

## Results

### Size, morphology and protein entrapment

**Figure 2:**
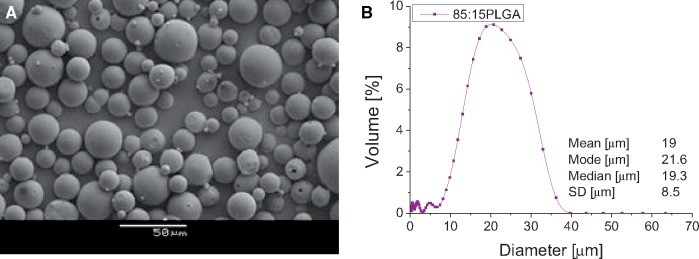
Representative SEM micrograph (**A**) and microparticles size distribution (**B**) of PLGA 85:15 microparticles.

The FITC-BSA loading percentage was 1% for both batches. Figure 2A shows a representative image of the surface morphology of the microparticles produces from water in oil in water (w/o/w) emulsion method using PLGA 85:15. The microparticles have a smooth, spherical and largely non-porous morphology. A typical size distribution of the PLGA 85:15 microparticles is shown in Fig. 2B. The mean microparticle size was 19 ± 8.5 μm.

**Figure 3:**
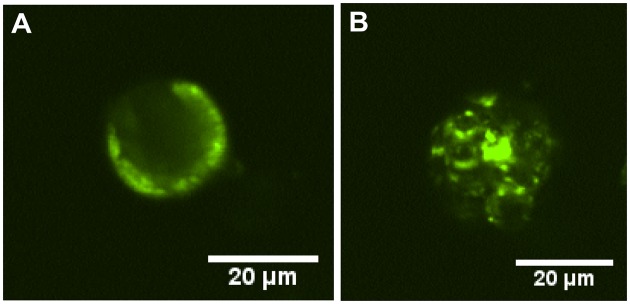
CLSM cross section of PLGA 85:15 microparticles showing the protein distribution inside the microparticles: (**A**) for the MP-1 batch and (**B**) for the MP-2 batch microparticles.

The fluorescence distribution within the microparticles from each batch is displayed in Fig. 3. In the MP-1 batch, the FITC-BSA is localized near the surface of the microparticles (Fig. 3A). In contrast, the FITC-BSA within the microparticles from the MP-2 batch is distributed within the depth of the microparticles apparently partially within protein-loaded cavities (Fig. 3B).

### Incorporating of PLGA 85:15 microparticles into 3D ice-templated scaffolds

**Figure 4:**
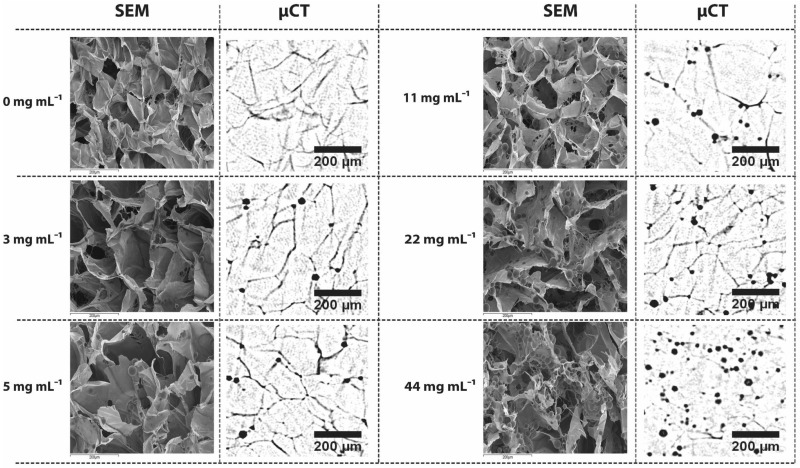
Morphological architecture of 3D ice-templated collagen scaffolds loaded with PLGA 85:15 microparticles at various concentrations. Representative SEM images and two-dimensional μCT data set showing the distribution of PLGA 85:15 microparticles within 3D ice-templated collagen scaffolds (SEM magnification ×250 and scale bar 200 μm).

To assess the effect of microparticles addition on scaffold architecture, 3D ice-templated collagen scaffolds loaded with PLGA 85:15 microparticles loaded with FITC-BSA were studied. μCT and SEM, were used to reveal the distribution of the PLGA 85:15 microparticles within the 3D ice-templated collagen scaffold (Fig. 4), and to evaluate the internal structure of the 3D scaffolds.

**Figure 5:**
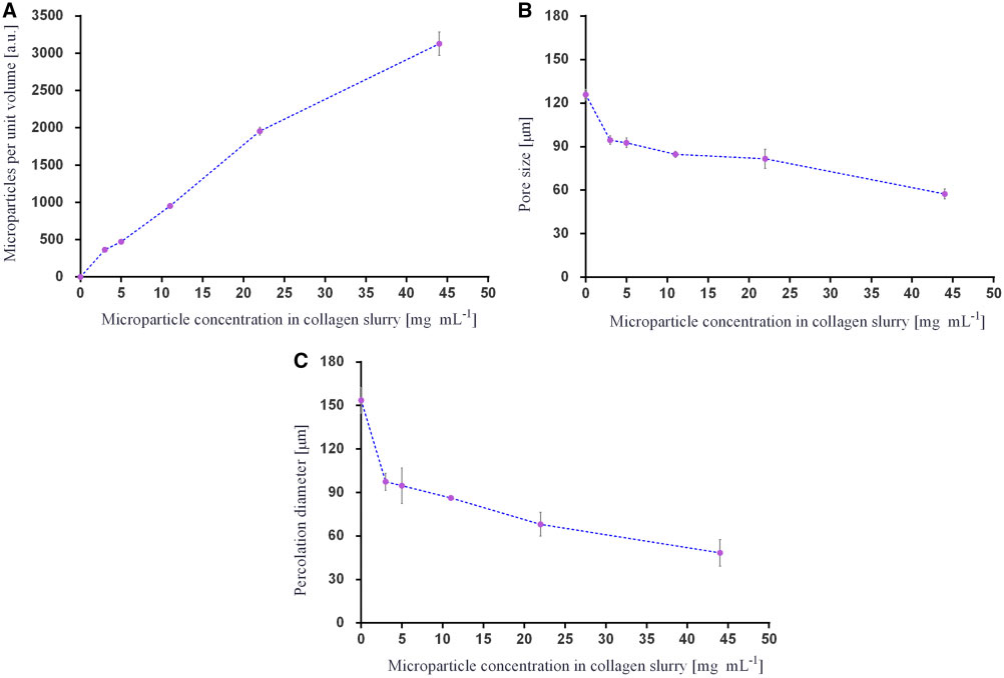
Results of microCT analysis: (**A**) total number of PLGA 85:15 microparticles per unit volume (*n* = 3); (**B**) pore size analysis (*n* = 4); and (**C**) percolation diameter (n = 4). Data are represented as mean ± SD.

Using the reconstructed 3D images from μCT, the total number of microparticles per unit volume was acquired (Fig. 5A) and the mean pore size and percolation diameter of the scaffolds evaluated (Fig. 5B and C). The addition of PLGA 85:15 microparticles into the 3D ice-templated collagen scaffolds results in a drop in the percolation diameter and pore size.

### FITC-BSA release studies

**Figure 6:**
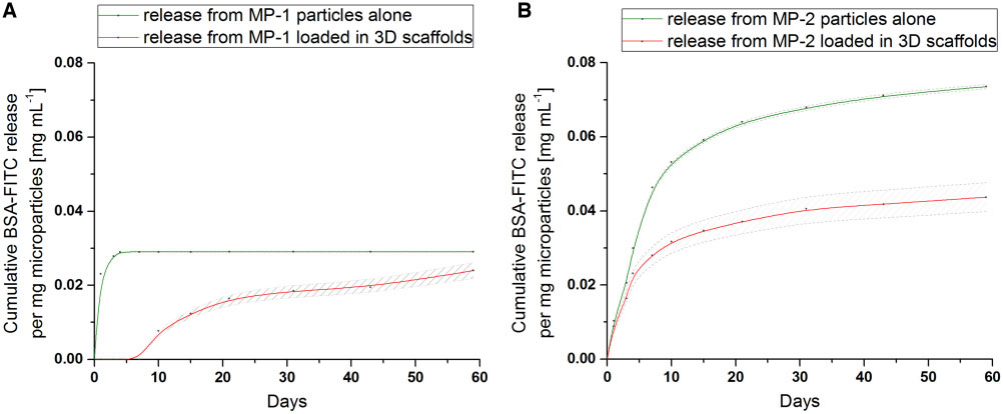
FITC-BSA release profile from MP-1 (**A**) and MP-2 (**B**) PLGA 85:15 microparticles before (green) and after (red) loading within the 3D ice-templated collagen scaffolds (dashed lines represent the standard deviation of each experiment).

Figure 6 shows the release profiles from the MP-1 and MP-2 microparticles alone (i.e. when not incorporated into the scaffold) (green profile), and from the microparticles incorporated in the crosslinked scaffolds (red profile) over 60 days *in vitro* (Fig. 6). The MP-1 microparticles alone release more quickly than those from MP-2 alone, and have a lower overall loading.

Once loaded and crosslinked within the ice-templated collagen scaffolds, the MP-1 microparticles (red profile) exhibit a 7-day delay in release (Fig. 6A). In contrast, the MP-2 microparticles loaded in the collagen scaffolds (red profile) begin release immediately with no delay (Fig. 6B).

## Discussion

The scaffolds displayed a homogeneous distribution of PLGA 85:15 microparticles at all loading levels studied. Increasing the microparticle concentration from 3 to 44 mg⋅ml^−1^ in the slurry resulted in a decrease in both mean pore size and percolation diameter. The increasing number of microparticles within the 3D ice-templated collagen structure changes the architecture of the struts and by default the dimensions of the pores inside the scaffolds. This modifies the pathway within the 3D ice-templated collagen scaffolds by reducing the available volume of the pores. However, the minimum percolation diameter observed in this study of 60 μm is larger than the threshold value reported by Ashworth *et al*. [53] below which cell invasion was significantly inhibited for periodontal ligament fibroblasts. This suggests that cell invasion should not be unduly restricted by the changes in pore morphology found with any of the microparticle additions reported here. It is likely that the diffusion of the FITC-BSA to the exterior solution will be slowed slightly by the solid fraction of the scaffold, but again, any effects from the small differences in scaffold structure resulting from microparticle addition are likely to be minimal.

The release profile of FITC-BSA from the microparticles alone was strongly correlated with the internal protein distribution. FITC-BSA release from the MP-1 microparticles was rapid, reaching a plateau after 4 days (Fig. 6A). This is consistent with the location of the majority of the protein at the surface of the microparticles (Fig. 3A). In contrast, the MP-2 microparticles gave a slow release continuing over 60 days (Fig. 6A). In these microparticles, the protein is located within the internal bulk of the microparticles (Fig. 3B) leading to the longer release time.

As seen in Fig. 6B, the release from the microparticles embedded within the crosslinked scaffolds differed from that of the microparticles in isolation. When within the scaffold, MP-1 and MP-2 microparticles, release less FITC-BSA than the microparticles alone. The release from the crosslinked scaffolds containing MP-1 was delayed by 7 days, but no such delay in release was observed for the scaffolds containing MP-2. Hence the protein distribution in the microparticles influences the effect of crosslinking on release.

EDC crosslinking involves the use of lysine (Lys, K) residues-free primary amino groups and carboxylate groups from glutamate (Glu, E) and aspartate (Asp, D) residues. Glutamate can be found in the GFOGER motif within triple-helical collagen structure, and aspartate in the RGD sequence of gelatine, both identified as high-affinity integrin binding sites [55–57]. The use of such amino acids during crosslinking can affect the integrin binding sites and consequently *in vitro* cell behaviour on collagen-based materials [58, 59]. A series of studies confirmed that the crosslinking of collagen-based materials is crucial in achieving suitable mechanical properties but, at the same time alters the cell response [11, 58]. The ice-templated collagen scaffolds loaded with PLGA microparticles (MP-1 and MP-2) were crosslinked at a molar ratio of 0.5:0.2:1 of EDC:NHS relative to the collagen carboxylic acid groups, defined as 10% crosslinking condition. The crosslinking percentage was chosen based on previous work indicating that a lower crosslinking levels will retain more native-like integrin engagement binding sites [58].

EDC/NHS crosslinking has been shown to improve resistance to degradation of collagen more effectively than dehydrothermal treatments. The use of EDC/NHS to crosslink collagen-based materials prove an increase enzymatic resistance, upregulation of proinflammatory and angiogenic factors *in vitro* [60–62]. *In vivo* collagen-based materials crosslinked by EDC/NHS reveal good cell infiltration, scaffold degradation and remodelling [63–65].

While crosslinking of the collagen scaffold is beneficial, the protein active agent within the microparticles may also take part in the reaction. Like collagen, FITC-BSA contains Lys, Asp and Glu residues [55]. As a consequence, the FITC-BSA from the scaffold embedded PLGA 85:15 microparticles is strongly affected by the crosslinking process.

**Figure 7:**
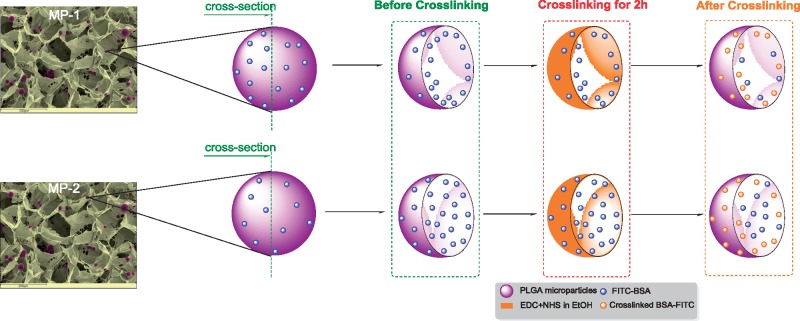
Schematic action mechanism of the EDC on the FITC-BSA within the PLGA 85:15 microparticles batches (MP-1 and MP-2).

Figure 7 shows the proposed mechanism of action of EDC on the MP-1 and MP-2 PLGA 85:15 microparticles. Under the conditions studied, the 95% ethanol-based crosslinking solution containing EDC and NHS acts only at the surface regions of the microparticles and does not penetrate the full bulk. Only protein present at or near the surface of the microparticles is affected by the reaction. Since the MP-1 microparticles have more surface protein, more is crosslinked and inactivated, delaying and reducing the overall release. In MP-2, more of the protein is protected within the bulk of the microparticles and no delay in release is seen.

## Conclusions

We have shown that protein-loaded PLGA 85:15 microparticles may be incorporated within ice-templated collagen scaffolds with homogeneous distribution. The process reduces the dimensions of both the pores and the percolation diameter of the scaffolds, but these parameters did not fall below those reported as necessary for cell invasion.

The application of EDC crosslinking, routinely used to stiffen and stabilize collagen scaffolds influences the release from the microparticles. Protein located within the bulk of the microparticles is protected from the crosslinking reaction and demonstrated a release profile that was both immediate and sustained. The protein distribution inside the microparticles therefore plays a crucial role in determining the drug delivery profile and its response to the crosslinking. These data may be of clinical utility in the design of scaffolds combining microparticles and bioactive molecules for sustained molecular and regenerative therapies.

## Supplementary data

Supplementary data are available at *REGBIO* online.

## Funding

This work was supported by the European Research Council [ERC Advanced Grant 320598 3D-E] and was also funded by a grant from the Medical Research Council, Arthritis Research UK, Reumafonds and the UKRMP. All Supporting Research Data according to ERC policy can be accessed at https://doi.org/10.17863/CAM.37794.

*Conflict of interest statement*. None declared.

## Supplementary Material

rbz015_Supplementary_Data
