# Supplementary material for: Targeted protein delivery: carbodiimide crosslinking influences protein release from microparticles incorporated within collagen scaffolds
Source: Regen Biomater. 2019 Apr 22;6(5):279–87. doi: 10.1093/rb/rbz015 (PMC6783698; doi:10.1093/rb/rbz015)
Supplement: rbz015_Supplementary_Data [file rbz015_supplementary_data.zip › rbz015-Suppl_data/Supplementary Information.docx]

**Supplementary Information**

**Targeted protein delivery: carbodiimide crosslinking influences protein release from microparticles incorporated within collagen scaffolds**

C. E. Tanase^1^, O. Qutachi^2†^, L. J. White^2^, K. M. Shakesheff^2^, A.W. McCaskie^3^, S. M. Best^1^, R. E. Cameron^1^

^1^Cambridge Centre for Medical Materials, Department of Materials Science and Metallurgy, University of Cambridge, CB3 0FS United Kingdom

^2^School of Pharmacy, University of Nottingham, NG7 2RD, United Kingdom

^3^Division of Trauma & Orthopaedic Surgery, Department of Surgery, University of Cambridge CB2 0QQ, United Kingdom

^†^*Present address*: School of Pharmacy, De Montforte University, LE1 9BH, United Kingdom

[cetanase@cantab.net](mailto:cetanase@cantab.net)

Methods:

To obtain release profiles from the PLGA 85:15 microparticle batches, 25 mg microparticles from MP-2 were dispersed into 1.5 mL of crosslinking solution (EDC/NHS) for 2 hours. The crosslinking solution was used in a range from 0 to 100%. Where 100% crosslinking solution was defined as a molar ratio of EDC to NHS to COOH of 5:2:1 using ethanol (95% v/v) as solvent. After the incubation time the particles were washed with PBS by centrifugation and resuspended in 1.5mL of PBS and placed in an incubator at 37 °C under shaking at 10 rpm for the entire duration of the experiment (e.g. 80 days). 100 μL from the supernatant was collected at the prescribed time points and analysed for FITC-BSA content. The same volume of fresh PBS was replenished into the microtubes, simulating the constant dissipation of the released drug inside the body.

The effect of the crosslinking solution (EDC/NHS) on the release of the FITC-BSA from the microparticles alone. Figure S1 indicates that the EDC/NHS lowers the rate of release of FITC-BSA from microparticles the effect increasing with increasing crosslinking level. This data is consistent with our hypothesised mechanism of action of the crosslinking solution on the FITC-BSA within the PLGA 85:15 microparticles (Figure 7).

It is interesting to note that the overall level of release of FITC-BSA from the MP-2 particles embedded within the scaffolds is lower than that seen in from the MP-2 microparticles alone. This may be the result of a degree of protein binding to the scaffold (Figure 6).


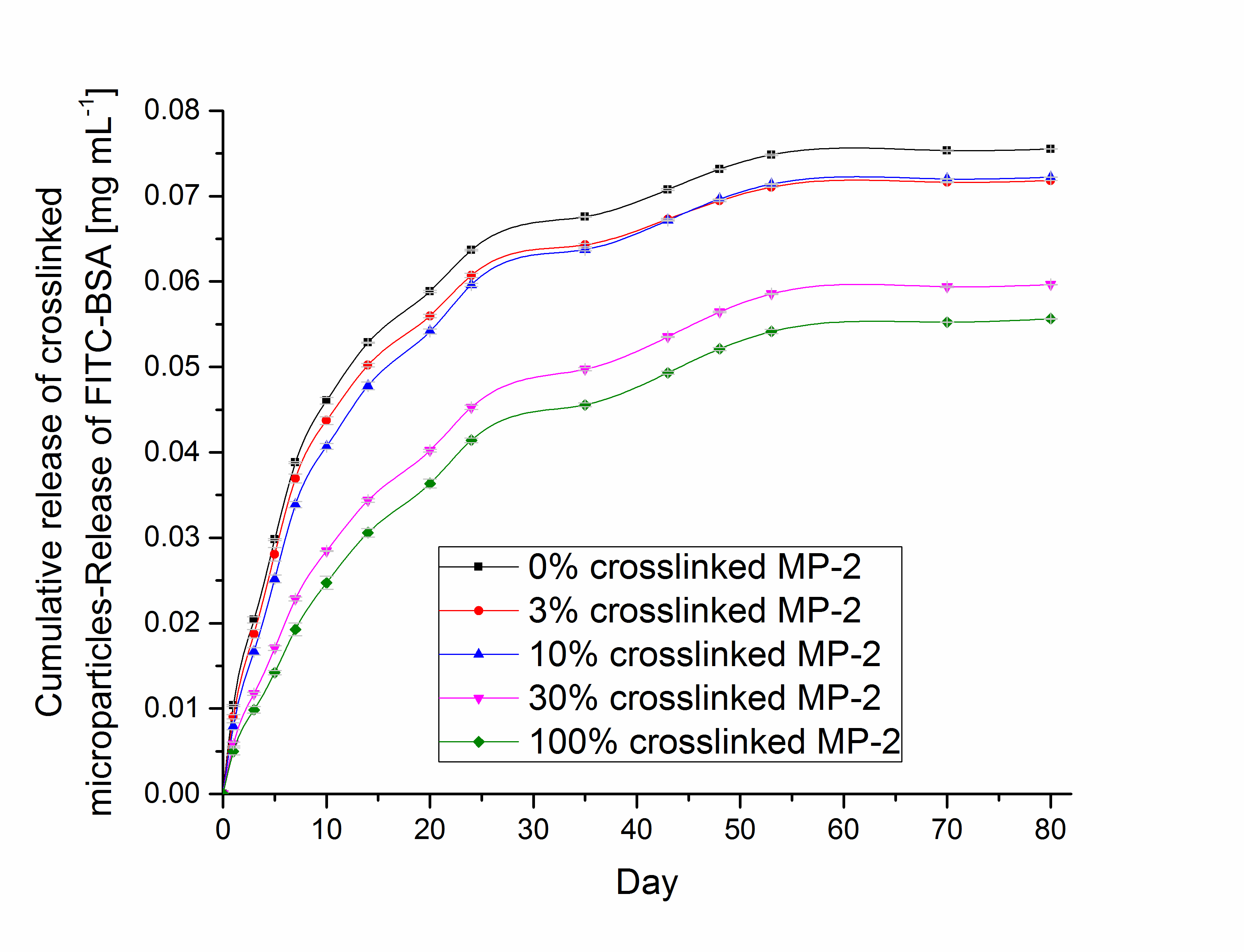


Figure S1. Effect of EDC/NHS on the drug delivery profile of FITC-BSA from PLGA 85:15 microparticles.
